# Changes in Bone Parameters and Serum Zinc Levels Following Oral Zinc Supplementation in Duchenne Muscular Dystrophy: A Quasi-Experimental Study

**DOI:** 10.3390/ijerph23060812

**Published:** 2026-06-18

**Authors:** Thaís Borges, Evellyn Grilo, Thais Alves Cunha, Luana Lima, Karina Vermeulen-Serpa, Mário Dourado-Júnior, Marília Lopes, Núbia Torres, Breno Bezerra, José Brandão-Neto, Sancha Vale

**Affiliations:** 1 Postgraduate Program of Health Sciences, Federal University of Rio Grande do Norte, Natal 59078-970, Brazil; thaisldborges@gmail.com (T.B.); evellyn-cg@hotmail.com (E.G.); thaisalvesc1@gmail.com (T.A.C.); karinavermeulen@hotmail.com (K.V.-S.); brandao-neto@live.com (J.B.-N.); 2 Department of Nutrition, Federal University of Rio Grande do Norte, Natal 59078-970, Brazil; luanalima.102@gmail.com (L.L.); marilia.lopes@ufrn.br (M.L.); 3 Department of Integrated Medicine, Federal University of Rio Grande do Norte, Natal 59078-970, Brazil; medourado03@gmail.com; 4 Postgraduate Program in Nutrition, Federal University of Rio Grande do Norte, Natal 59078-970, Brazil; rafaellamoreira@hotmail.com; 5 Primary Processing and Reuse of Produced Water and Wastewater Center, Federal University of Rio Grande do Norte, Natal 59078-970, Brazil; brenogpb@gmail.com; 6 Department of Clinical Medicine, Federal University of Rio Grande do Norte, Natal 59078-970, Brazil

**Keywords:** muscular dystrophies, dietary supplements, bone density

## Abstract

**Highlights:**

**Public health relevance—How does this work relate to a public health issue?**

- Individuals living with Duchenne muscular dystrophy (DMD) are vulnerable to nutritional imbalances that may compromise bone health and long-term health outcomes.
- Monitoring micronutrient status is important for the comprehensive clinical and nutritional care of populations affected by rare neuromuscular diseases.

**Public health significance—Why is this work of significance to public health?**

- Zinc deficiency was identified in a substantial proportion of individuals with DMD, indicating a potential nutritional vulnerability in this population.
- Oral zinc supplementation increased serum zinc concentrations among participants with baseline zinc deficiency and was associated with modest improvements in bone parameters in a subgroup of participants.

**Public health implications—What are the key implications or messages for practitioners, policy makers and/or researchers in public health?**

- Monitoring serum zinc concentrations may contribute to the nutritional assessment of individuals with DMD.
- Nutritional strategies addressing micronutrient status may support broader bone health management in individuals living with chronic neuromuscular diseases.

**Abstract:**

Individuals with Duchenne muscular dystrophy (DMD) are prone to nutritional imbalances, and zinc deficiency may contribute to impaired bone health. This study evaluated serum zinc status and the effects of oral supplementation on bone parameters in DMD. In this quasi-experimental before-and-after study, 34 patients were assessed at three time points over eight months. Eligible participants who met the inclusion criteria and agreed to participate received the proposed interventions during routine follow-up at the Neurology outpatient clinic. Anthropometry, dietary intake, bone mineral density (BMD), bone mineral content (BMC), and serum zinc were measured; supplementation (5–15 mg/day) was provided for four months. Baseline zinc deficiency was observed in 36.7% of participants. No significant overall changes were detected. Stratified analyses revealed a modest increase in total body BMD among individuals with adequate baseline BMD (*p* = 0.02). As this finding emerged from a subgroup analysis, it should be interpreted cautiously, and the potential contribution of physiological growth to the observed change cannot be excluded. In addition, zinc-deficient participants showed a significant rise in serum zinc levels (*p* = 0.008). These findings suggest that the response to zinc supplementation may vary according to baseline nutritional and skeletal status and underscore the relevance of micronutrient monitoring in individuals with DMD. Trial registration: The trial was also registered in the Brazilian Registry of Clinical Trials under the code RBR-7cfdxm, approved on 14 June 2018.

## 1. Introduction

Duchenne muscular dystrophy (DMD) is a rare genetic disorder caused by mutations in the DMD gene, which encodes dystrophin, a protein essential for skeletal and cardiac muscle integrity. The absence of dystrophin results in progressive muscle degeneration, chronic inflammation, and loss of muscle function, leading to severe disability and multisystem complications [1,2,3].

Although DMD is considered a rare disease, individuals living with this condition represent a nutritionally and clinically vulnerable population. Disease progression, reduced mobility, chronic inflammation, and long-term pharmacological treatment may negatively affect growth, body composition, and bone health. These factors contribute to an increased risk of nutritional imbalances and metabolic complications, which may further compromise quality of life and health outcomes [4,5].

Glucocorticoid therapy is currently the standard treatment for DMD, as it delays the progression of muscle weakness and improves respiratory, cardiac, and functional outcomes. However, long-term use of glucocorticoids is associated with several adverse effects, including growth impairment, delayed puberty, weight gain, and reduced bone mineral density, which increases the risk of fractures [4,5]. Consequently, strategies aimed at preserving bone health are an important component of the clinical management of individuals with DMD.

Bone fragility in this population is influenced not only by pharmacological treatment and reduced mobility but also by nutritional factors. Inadequate intake or deficiency of micronutrients involved in bone metabolism may contribute to impaired bone health. Calcium and vitamin D are commonly recommended to support bone health in individuals receiving glucocorticoid therapy; however, other micronutrients may also play relevant roles in skeletal metabolism [6,7].

Among these micronutrients, zinc has been recognized as an important regulator of bone metabolism and is also involved in pathways relevant to muscle physiology and inflammation. Experimental and clinical evidence suggests that zinc may stimulate osteoblastic activity, reduce bone resorption, and modulate inflammatory and hormonal pathways involved in bone remodeling [6,7]. Zinc deficiency has been associated with decreased bone mineral density and impaired skeletal development in different populations [8].

Despite the potential role of zinc in bone metabolism, evidence regarding the effects of zinc supplementation on bone health in individuals with Duchenne muscular dystrophy remains limited. The rarity of the condition poses substantial challenges for clinical and nutritional research, including limited sample sizes and restricted opportunities for randomized controlled trials. Consequently, observational and quasi-experimental designs are frequently employed to investigate potential nutritional interventions in this population.

In this context, investigating nutritional determinants of bone health may contribute to improving the management of individuals with Duchenne muscular dystrophy. Therefore, this study aimed to evaluate changes in serum zinc concentrations and bone parameters following oral zinc supplementation in individuals with DMD. We hypothesized that zinc supplementation would increase serum zinc concentrations and may be associated with favorable changes in selected bone parameters.

## 2. Materials and Methods

### 2.1. Study Design and Ethical Considerations

This quasi-experimental before-and-after study was conducted at the Neurology outpatient clinic of the Onofre Lopes University Hospital (HUOL), Natal, Brazil, between February 2018 and March 2020. The study was designed, conducted, and reported in accordance with the Transparent Reporting of Evaluations with Nonrandomized Designs (TREND) Statement Checklist (Table S1) [9]. The study was approved by the Research Ethics Committee of HUOL (Approval No. 1,754,017) in September 2016 and was subsequently registered in the Brazilian Registry of Clinical Trials (ReBEC; identifier RBR-7cfdxm) in June 2018. Trial registration was therefore completed retrospectively. Written informed consent was obtained from the legal guardians of all participants prior to enrollment. 

### 2.2. Participants and Eligibility Criteria

Patients with DMD eligible for this study were routinely followed at the Neurology outpatient clinic of the Onofre Lopes University Hospital (HUOL). Inclusion criteria comprised age ≥5 years and a diagnosis of DMD confirmed by clinical history and genetic testing. Patients using medications for the treatment of osteoporosis or supplements containing zinc were excluded. Individuals who discontinued the intervention or did not complete any of the baseline (T0) assessments were also excluded.

**Figure 1:**
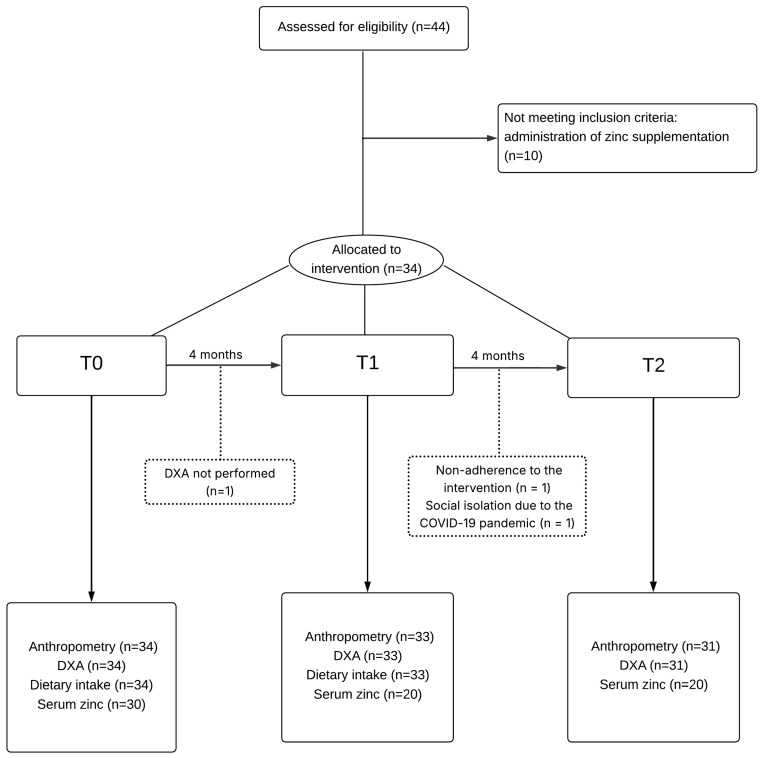
Flow diagram illustrating the progress of participants through the phases.

Given the rarity of DMD and the limited number of patients followed at the HUOL Neurology outpatient clinic during the study period (*n* = 44), no sampling procedure was performed. Of these, 34 individuals met the eligibility criteria and were included in the study (Figure 1).

### 2.3. Interventions

All study interventions and follow-up assessments were performed by the research team at the Onofre Lopes University Hospital (HUOL). At baseline (T0), the study population was initially characterized. Assessments were conducted at three distinct time points: T0 (baseline), T1 (pre-supplementation, approximately four months after baseline), and T2 (post-supplementation, approximately four months after T1).

Zinc supplementation was implemented during the period between T1 and T2. All participants received oral zinc supplementation in the form of zinc bisglycinate chelate, provided as a liquid formulation and administered once daily (single shot) in the morning, while concomitantly maintaining routine outpatient supplements, including calcium (500 mg/day) and vitamin D (400 IU/day). Patients and their families were instructed on the appropriate storage of the zinc supplement, which was to be kept in a well-ventilated location protected from direct sunlight.

The daily zinc dose was adjusted according to the participants’ age group: 5 mg for those aged 5 to 8 years, 10 mg for those aged 9 to 13 years, and 15 mg for individuals aged 14 years or older. Dose selection was based on previously published estimates of dietary zinc intake among Brazilian children [10]. Although mean dietary zinc intake exceeded the Estimated Average Requirement, supplementation was provided to all participants because biochemical assessment indicated a substantial prevalence of serum zinc deficiency in the study population, and dietary intake estimates may not fully reflect zinc status, particularly in individuals with Duchenne muscular dystrophy. These doses were intended to supplement habitual dietary intake and are within the range used in pediatric zinc supplementation studies aimed at improving zinc status. The supplementation protocol was designed to ensure that total zinc intake, considering both dietary intake and supplementation, remained below the tolerable upper intake level established by the Institute of Medicine [11].

Between T1 and T2, zinc shots were individually dispensed to each participant on two separate occasions, in packages containing 60 shots each, totaling 120 units per participant. Adherence to the intervention was monitored through weekly telephone contacts with participants’ legal guardians and by weekly recording of the remaining zinc shots based on photographic documentation of unused units. Adherence to zinc supplementation was 100% among participants who completed the intervention period, as confirmed by these monitoring procedures.

### 2.4. Anthropometric Assessment

Anthropometric measurements, including body weight (kg) and height (m), were obtained using a digital scale with an attached stadiometer. For the participant who used a wheelchair, body weight was measured using a wide-platform electronic scale, and height was measured with the individual in the supine position. These measurements were used to calculate body mass index (BMI).

Anthropometric assessment of children and adolescents was based on the classification of z-scores for the height-for-age, weight-for-age, and BMI-for-age indices. For adults (>19 years), BMI was used for nutritional status classification [12].

### 2.5. Assessment of Bone Mineral Density and Bone Mineral Content

Bone mineral density (BMD) and bone mineral content (BMC) were measured by dual-energy X-ray absorptiometry (DXA) using a Lunar DPX-NT system. Lunar version 4.7, General Electric Company, Waukesha, WI, USA was used for participants aged 5–20 years and adult software for those older than 20 years. All examinations were performed by a properly trained technician, with patients positioned in the supine position, wearing light clothing free of metallic materials, in accordance with current recommendations [13].

Lumbar spine (L1–L4) and total body BMD were expressed in g/cm^2^ and as z-scores. Total body BMD was considered low when the z-score was ≤−2, according to the criteria of the International Society for Clinical Densitometry [14]. Bone mineral content was expressed in kilograms (kg), as reported by the Lunar DPX-NT software (version 4.7, General Electric Company, Waukesha, WI, USA).

### 2.6. Assessment of Serum Zinc

A 3 mL sample of venous blood was collected in the morning after a 12-h overnight fast for the determination of serum zinc concentration using 3.5 mL vacuum tubes containing a separator gel and clot activator. Sample collection and processing followed standardized procedures designed to minimize the risk of hemolysis. After a minimum resting period of 30 min, samples were centrifuged for 10 min at 3400 rpm. Aliquots of 500 µL of serum were transferred to 1.5 mL polypropylene tubes and stored at −80 °C, together with the remaining serum. All materials used for sample collection, handling, and storage were metal-free to prevent contamination.

Serum samples were thawed and subjected to pretreatment for zinc determination. Aliquots of 0.4 mL of serum were transferred to tubes, followed by the addition of 0.4 mL of nitric acid and 3.2 mL of ultrapure water, and then homogenized for 2 min using a vortex mixer.

Serum zinc concentrations were determined by inductively coupled plasma optical emission spectrometry (ICP-OES) using an iCAP 6300 Duo instrument (Termo Fisher Scientific, Bremen, Germany) measured following previously validated procedures.

Reference values for serum zinc concentration in male individuals were 65 µg/dL for those aged <10 years and 70 µg/dL for those aged ≥10 years. In addition, it is recommended that when 20% of the evaluated population presents serum zinc concentrations below these values, the population be considered at risk of zinc deficiency [15].

### 2.7. Dietary Assessment

Habitual dietary intake was assessed using two 24-h dietary recalls (24HR), administered at T0 and T1 by trained researchers, with one recall referring to a weekday and the other to a weekend day. Patients and their caregivers reported all foods and preparations consumed on the previous day, specifying the type of food, quantities using household measures, cooking methods, and, in the case of industrialized products, the brand. Prior to analysis, the researchers developed a standardized list of foods and preparations. Energy intake, macronutrients (proteins, carbohydrates, and lipids), fiber, and micronutrients relevant to bone health, including calcium, vitamin D, and zinc, were evaluated.

After individual analysis of the raw data, the percentages of inadequate intake of energy, macronutrients, and fiber were calculated based on the Acceptable Macronutrient Distribution Range (AMDR) values proposed by the Institute of Medicine, considering a sedentary level of physical activity for the estimation of energy requirements [16].

Intrapersonal variability between the two 24HR was adjusted using the Multiple Source Method to estimate micronutrient intake, which was subsequently adjusted for total energy intake of each 24HR using SPSS^®^ software (version 28.0). Data were expressed as mean and standard deviation.

For the evaluated micronutrients, the prevalence of inadequate intake was estimated with adjustment for intrapersonal variability and energy intake, using *z* values calculated from the energy-adjusted mean and standard deviation and the Estimated Average Requirement (EAR). The *z* values were then applied to the normal distribution to obtain the percentage of inadequate intake for each micronutrient. Dietary intake analysis was stratified by age groups (4–8, 9–13, 14–18, and 19–30 years), in accordance with the Dietary Reference Intake (DRI) recommendations [11,17].

### 2.8. Statistical Analyses

Statistical analyses were performed using SPSS^®^ software (version 28.0). The distribution of continuous variables was assessed using the Shapiro–Wilk test, and homogeneity of variance was evaluated prior to the application of parametric analyses. As no significant deviations from normality were detected (all *p* > 0.05), data are presented as means and standard deviations, and parametric statistical methods were used throughout the analyses.

Initially, anthropometric variables, serum zinc levels, and bone parameters were compared across time points (T0, T1, and T2) for the entire group using one-way analysis of variance (ANOVA), followed by Bonferroni post hoc testing. This approach was selected to evaluate differences in mean values across the study time points. Differences were considered statistically significant when *p* < 0.05.

Subsequently, participants were categorized according to total body BMD *z*-scores at T1 into two groups: adequate BMD and low BMD. Age comparisons between the adequate-BMD and low-BMD groups were performed using the independent Student’s *t* test. Participants were also categorized at T1 into two groups based on serum zinc levels: with zinc deficiency and without zinc deficiency. T1 was selected for this categorization because it corresponded to the time point at which zinc supplementation was initiated.

## 3. Results

The flow of participants is illustrated in Figure 1. Among those assessed for eligibility, 10 were excluded due to recent use of zinc supplementation. At T1, one patient was excluded for not undergoing dual-energy X-ray absorptiometry (DXA) because of respiratory difficulties in the supine position (Figure 1).

During the zinc supplementation period (between T1 and T2), one participant was lost to follow-up, which precluded the performance of anthropometric and DXA assessments for these individuals at T2. With regard to the analysis of serum zinc concentrations, 13 participants did not undergo blood collection, resulting in a sample size of 20 individuals for this variable (Figure 1).

### 3.1. Baseline Characterization of Participants (T0)

The mean age of participants was 12.6 ± 5.0 years, ranging from 5.0 to 24.2 years. All participants aged 5 to 10 years (*n* = 12) had adequate weight-for-age z-scores. Height-for-age and BMI-for-age assessments were performed in 28 participants aged 5 to 19 years, of whom 39.3% (*n* = 11) presented with short stature. Regarding BMI-for-age, 14.3% (*n* = 4) were classified as underweight, 42.8% (*n* = 12) as having normal weight, 28.6% (*n* = 8) as overweight, and 14.3% (*n* = 4) as obese. Adult participants (*n* = 6) were classified according to BMI as underweight (33.3%; *n* = 2), normal weight (50.0%; *n* = 3), and overweight (16.7%; *n* = 1).

At baseline (T0), serum zinc concentrations were assessed in 30 participants, of whom 36.7% (*n* = 11) had values below the reference limits. With respect to bone parameters, 41.2% (*n* = 14) of the evaluated individuals presented with low lumbar spine bone mineral density (BMD), while 38.2% (*n* = 13) had low total body BMD. In addition, among the 34 participants included in the analysis, 88.2% (*n* = 30) were receiving glucocorticoid therapy (prednisone), 73.5% (*n* = 25) were using calcium supplementation, and 82.4% (*n* = 28) were receiving vitamin D supplementation (Table 1).

Regarding energy intake, 53.0% (*n* = 18) of participants exhibited intake below the recommended range, while 44.1% (*n* = 15) exhibited intake above the recommended range, indicating that most participants presented energy intake outside the recommended range. With respect to protein intake, 100% demonstrated adequate intake. The majority of participants (88.2%; *n* = 30) had adequate carbohydrate intake, and only 11.8% (*n* = 4) had intake above the recommended level. Regarding lipids, 41.2% (*n* = 14) had adequate intake, whereas 58.5% (*n* = 20) exhibited low lipid intake. Most participants showed low fiber intake (85.3%; *n* = 29), and 14.7% (*n* = 5) had adequate fiber intake.

Concerning the percentages of inadequacy in calcium, vitamin D, and zinc intake, high levels of inadequacy were observed for dietary calcium and vitamin D intake (>50%). In contrast, no high percentage of inadequacy was found for zinc intake in any age group (Table 2).

### 3.2. Anthropometric Variables Before and After Zinc Supplementation

In the analysis of the total group (*n* = 34), no statistically significant differences were observed in weight, height, or BMI across the three assessment time points (T0, T1, and T2) (*p* > 0.05). After stratifying participants into two groups according to bone mineral density (adequate BMD and low BMD), a statistically significant difference was identified between the mean ages of these groups (*p* = 0.0005). The group with adequate BMD had a mean age of 10.3 ± 4.4 years, whereas the group with low BMD had a mean age of 15.9 ± 3.8 years.

In the group with adequate bone mineral density (BMD) (*n* = 20), a statistically significant increase in weight and height was observed across the three assessment time points (T0, T1, and T2) (*p* < 0.05). BMI showed a significant increase between T0 and T1; however, no statistically significant differences were observed between T1 and T2 (*p* > 0.05) (Table 3).

In the group with low BMD (*n* = 14), no statistically significant differences were identified in anthropometric variables (weight, height, and BMI) across the three assessment time points (T0, T1, and T2) (*p* > 0.05) (Table 3).

### 3.3. Bone Parameters Before and After Zinc Supplementation

In the overall sample analysis (*n* = 34), no statistically significant differences were observed in bone parameters—bone mineral density (BMD) and bone mineral content (BMC)—across the three assessment time points (T0, T1, and T2) (*p* > 0.05).

Among participants with adequate BMD (*n* = 20), a statistically significant increase in BMC was observed between T0 and T1 (*p* < 0.05), as well as between T0 and T2 (*p* < 0.05). Additionally, a significant increase in total body BMD (g/cm^2^) was identified after zinc supplementation (*p* < 0.05) (Table 4).

In the group with low BMD (*n* = 14), no statistically significant differences were observed in bone parameters across the assessment time points (T0, T1, and T2) (*p* > 0.05) (Table 4).

### 3.4. Serum Zinc Concentrations Before and After Zinc Supplementation

In the overall sample analysis (*n* = 20), no statistically significant differences were observed in serum zinc levels across the three assessment time points (T0, T1, and T2) (*p* > 0.05).

**Figure 2:**
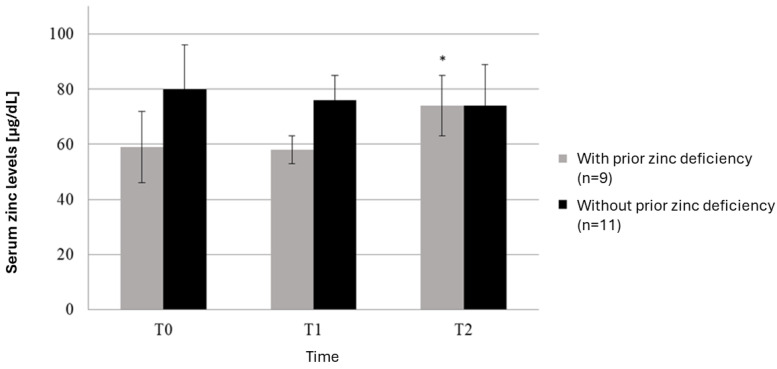
Effect of zinc supplementation on serum zinc levels in patients with Duchenne muscular dystrophy categorized according to baseline zinc status: zinc deficiency (*n* = 9) and no zinc deficiency (*n* = 11) (total *n* = 20). * Patients with baseline serum zinc deficiency exhibited a significant increase in zinc levels following supplementation (T1 to T2: 4-month zinc supplementation period, *p* = 0.008; T0 to T2: period including the no-intervention phase and the 4-month zinc supplementation period, *p* = 0.048). Test: One-way ANOVA with Bonferroni post hoc test.

After categorizing the study population according to the presence or absence of prior zinc deficiency, a statistically significant increase in serum zinc levels was observed in the subgroup with prior deficiency following supplementation (*p* < 0.05) (Figure 2). In this subgroup, the mean serum zinc concentrations were 59 ± 13 µg/dL at T0, 58 ± 5 µg/dL at T1, and 74 ± 11 µg/dL at T2.

Conversely, in the subgroup without prior zinc deficiency, no statistically significant differences in serum zinc levels were observed after supplementation (*p* = 0.359) (Figure 2). The mean concentrations in this subgroup were 80 ± 16 µg/dL at T0, 76 ± 9 µg/dL at T1, and 74 ± 15 µg/dL at T2.

## 4. Discussion

The present study investigated serum zinc status and the effects of oral zinc supplementation on bone parameters in individuals with Duchenne muscular dystrophy. Zinc supplementation increased serum zinc concentrations in participants with baseline deficiency and was associated with a modest increase in total body bone mineral density in those with preserved baseline bone density, whereas no significant changes were observed in the overall sample. These findings indicate that the response to zinc supplementation depends on baseline nutritional and skeletal status in individuals with DMD.

In this study, 41.2% and 38.2% of individuals exhibited low BMD at the lumbar spine and total body, respectively, which may be related to the high proportion of patients receiving glucocorticoid therapy [18]. Prolonged glucocorticoid use is associated with reduced bone mass and increased fracture risk [19], reinforcing the need for continuous bone health monitoring in this population.

Between T0 and T1, increases in weight, height, and BMI were observed only in participants with adequate BMD, consistent with expected growth in a predominantly pediatric sample. BMD remained unchanged in both groups following routine care with calcium, vitamin D, and glucocorticoids, in agreement with previous studies [20,21], whereas BMC increased in the adequate-BMD group, likely reflecting physiological growth, as also reported in supplemented DMD populations [22].

Zinc supplementation was associated with a modest increase in total body BMD in the adequate-BMD group, consistent with pediatric clinical trials [15,16]. No changes in BMD or BMC were observed in the low-BMD group despite calcium and vitamin D supplementation. This lack of improvement may reflect endocrine, metabolic, and immunological alterations linked to DMD and prolonged glucocorticoid use [23]. Patients with DMD exhibit impairment of the growth hormone–IGF-1 axis, reduced bone turnover, and elevated pro-inflammatory cytokines, which may compromise bone metabolism and attenuate responses to supplementation, particularly in older individuals with low BMD and prolonged glucocorticoid exposure [21,23]. Although statistically significant, the observed increase in total body BMD was small, and its clinical relevance should be interpreted with caution given the short intervention period and the absence of least significant change (LSC) data for the DXA measurements. In contrast, zinc supplementation significantly increased serum zinc levels in deficient individuals, indicating that response depends on baseline status, with enhanced absorption and adaptive regulation under deficiency [24].

However, the discrepancy between the relatively low prevalence of dietary zinc inadequacy and the high frequency of low serum zinc concentrations warrants consideration. Chronic inflammation, a hallmark of DMD, may alter zinc metabolism and promote zinc redistribution from the circulation to other tissues. In addition, long-term glucocorticoid therapy may affect zinc homeostasis and contribute to lower circulating zinc concentrations. These mechanisms may help explain the observed dissociation between dietary zinc intake and serum zinc status in this population.

No effect of zinc supplementation on anthropometric parameters was observed in either group, consistent with previous literature [15], and possibly influenced by endocrine disturbances associated with DMD, including impairment of the growth hormone–IGF-1 axis [25].

A high prevalence of nutritional alterations was also observed. Short stature was common, consistent with previous reports in DMD populations [26,27], and weight extremes, including overweight, obesity, and undernutrition, were frequent [28]. Inadequate intake of micronutrients essential for bone health was marked, with calcium inadequacy ranging from 88.1% to 99.5% and universal inadequacy of vitamin D intake. Although dietary zinc inadequacy was less frequent, 36.7% of participants had low serum zinc concentrations [14], indicating risk of deficiency.

The intervention was implemented within the routine outpatient follow-up setting, supporting the practicality of micronutrient supplementation in individuals with DMD. Adherence to supplementation and scheduled assessments was generally maintained despite challenges inherent to long-term follow-up in patients with progressive physical limitations and complex pharmacological management. Integration of the intervention into routine care may have contributed to participant retention and consistency of follow-up procedures. Nevertheless, barriers such as disease progression, mobility restrictions, and dependence on caregivers may have influenced adherence and partially attenuated the observed effects.

Although conducted in a single-center outpatient setting, this study reflects real-world clinical care in individuals with DMD, supporting the potential applicability of oral zinc supplementation in similar contexts. However, differences in disease severity, healthcare practices, and adherence may limit the generalizability of the findings to other populations and settings.

Limitations of this study include the small sample size due to the rarity of DMD, the quasi-experimental design without a concurrent control group, and the lack of hormonal and bone biomarker measurements. Although the study included a pre-intervention observation period, allowing participants to serve as their own reference over time, the potential influence of physiological growth and other time-dependent factors on the observed changes cannot be excluded. In addition, relevant clinical factors that may influence bone health, such as ambulatory status, glucocorticoid exposure, fracture history, pubertal stage, and disease severity, were not available for analysis. Furthermore, because all participants continued calcium and vitamin D supplementation throughout the study period, the independent contribution of zinc to the observed bone outcomes cannot be fully isolated. Despite these limitations, this study contributes to the limited evidence regarding zinc supplementation in individuals with DMD.

## 5. Conclusions

In this quasi-experimental study, oral zinc supplementation increased serum zinc concentrations in individuals with Duchenne muscular dystrophy presenting baseline deficiency and was associated with a modest increase in total body bone mineral density in those with preserved baseline bone density. These findings indicate that the effects of zinc supplementation are not uniform and depend on baseline nutritional and skeletal status. Monitoring micronutrient status, particularly zinc, may therefore represent a relevant component of clinical and nutritional assessment in this population. Further studies with larger samples and controlled designs are needed to clarify the role of zinc supplementation as a targeted strategy for bone health management in Duchenne muscular dystrophy.

## Figures and Tables

**Table 1:** Baseline demographic, anthropometric, biochemical, and bone characteristics of participants with Duchenne muscular dystrophy (T0) (*n* = 34).

Variables	Descriptive Statistics ^⁋^
Age (years)	12.6 ± 5.0
Body weight (kg)	37.3 ± 15.7
Weight-for-age (*z*-score) ^†^	−0.34 ± 0.89
Height (m)	1.37 ± 0.16
Height-for-age (*z*-score) ^‡^	−1.39 ± 1.40
BMI (kg/m^2^)	19.37 ± 5.56
BMI-for-age (*z*-score) ^‡^	0.10 ± 2.56
Serum zinc (µg/dL)	72.2 ± 18.3
Lumbar spine BMD (g/cm^2^)	0.69 ± 0.18
Lumbar spine BMD (*z*-score)	−1.66 ± 1.57
Total body BMD (g/cm^2^)	0.85 ± 0.08
Total body BMD (*z*-score)	−1.39 ± 1.18
Use of glucocorticoids [*n*; (%)]	30; (88.2)
Use of calcium supplementation [*n*; (%)]	25; (73.5)
Use of vitamin D supplementation [*n*; (%)]	28; (82.4)

**^⁋^** Mean ± standard deviation; ^†^ classification for patients aged 5 to 10 years (*n* = 12); ^‡^ classification for patients aged 5 to 19 years (*n* = 28). BMI = body mass index; BMD = bone mineral density.

**Table 2:** Nutritional recommendations, habitual micronutrient intake, and prevalence of inadequate micronutrient intake in patients with Duchenne muscular dystrophy (*n* = 34).

Micronutrient	EAR	Mean ± SD *	% Inadequacy
Calcium (mg)			
4–8 years (*n* = 8)	800	612.9 ± 204.0	88.1
9–13 years (*n* = 16)	1100	484.2 ± 108.8	99.5
14–18 years (*n* = 4)	1100	720.3 ± 361.7	99.5
19–30 years (*n* = 6)	800	424.0 ± 117.1	88.1
Vitamin D (µg)			
4–8 years (*n* = 8)	10.0	2.5 ± 1.3	100.0
9–13 years (*n* = 16)	10.0	1.5 ± 0.7	100.0
14–18 years (*n* = 4)	10.0	2.3 ± 0.7	100.0
19–30 years (*n* = 6)	10.0	1.5 ± 0.4	100.0
Zinc (mg)			
4–8 years (*n* = 8)	4.0	9.3 ± 2.2	0.8
9–13 years (*n* = 16)	7.0	10.1 ± 2.1	11.9
14–18 years (*n* = 4)	8.5	10.7 ± 3.0	28.4
19–30 years (*n* = 6)	9.4	9.5 ± 3.3	41.7

Abbreviations: EAR (Estimated Average Requirement); * Mean intake from two 24-h dietary recalls, adjusted for intrapersonal variability and total energy intake [11,17].

**Table 3:** Effect of zinc supplementation on anthropometric parameters of patients with DMD, stratified by total body bone mineral density classification (*n* = 34).

Variables	Classification of Total Body Bone Mineral Density							
	Adequate BMD (*n* = 20)			*p*-Value	Inadequate BMD (*n* = 14)			*p*-Value
	T0	T1	T2		T0	T1	T2	
Weight (kg)	32.4 ± 15.7 ^a^	34.3 ± 16.2 ^b^	37.4 ± 16.6 ^c^	<0.001	43.4 ± 13.9 ^a^	43.7 ± 14.0 ^a^	44.1 ± 14.2 ^a^	0.400
Height (m)	1.28 ± 0.16 ^a^	1.30 ± 0.17 ^b^	1.33 ± 0.17 ^c^	<0.001	1.47 ± 0.08 ^a^	1.48 ± 0.08 ^a^	1.48 ± 0.07 ^a^	0.075
BMI (kg/m^2^)	18.9 ± 5.5 ^a^	19.6 ± 5.6 ^b^	20.5 ± 6.0 ^b^	0.002	19.9 ± 6.0 ^a^	20.0 ± 6.1 ^a^	20.1 ± 6.3 ^a^	0.825

BMD: Bone mineral density; BMI: body mass index. Data are expressed as mean ± standard deviation. T0 to T1: 4-month period without intervention; T1 to T2: 4-month period of zinc supplementation. Different superscript letters indicate a statistically significant difference between time points (intragroup analysis) (*p* < 0.05). Test: one-way ANOVA with Bonferroni post hoc test.

**Table 4:** Effect of zinc supplementation on bone parameters in patients with DMD, stratified by total body BMD classification (*n* = 34).

Bone Parameters	Classification of Total Body Bone Mineral Density							
	Adequate BMD (*n* = 20)			*p*-Value	Inadequate BMD (*n* = 14)			*p*-Value
	T0	T1	T2		T0	T1	T2	
Lumbar spine BMD (g/cm^2^)	0.66 ± 0.15 ^a^	0.66 ± 0.16 ^a^	0.65 ± 0.15 ^a^	0.379	0.71 ± 0.19 ^a^	0.71 ± 0.17 ^a^	0.71 ± 0.16 ^a^	0.879
Lumbar spine BMD (*z*-score)	−0.81 ± 1.23 ^a^	−0.79 ± 1.3 ^a^	−1.05 ± 1.26 ^a^	0.136	−2.54 ± 0.97 ^a^	−2.68 ± 0.77 ^a^	−2.83 ± 0.67 ^a^	0.226
Total body BMD (g/cm^2^)	0.83 ± 0.05 ^a^	0.83 ± 0.05 ^a^	0.84 ± 0.04 ^b^	0.020	0.87 ± 0.08 ^a^	0.87 ± 0.09 ^a^	0.87 ± 0.08 ^a^	0.980
Total body BMD (*z*-score)	−0.63 ± 0.92 ^a^	−0.67 ± 0.72 ^a^	−0.62 ± 0.77 ^a^	0.137	−2.47 ± 0.55 ^a^	−2.63 ± 0.58 ^a^	−2.75 ± 0.68 ^a^	0.996
BMC	1.98 ± 0.61 ^a^	2.08 ± 0.62 ^b^	2.11 ± 0.64 ^b^	0.002	2.34 ± 0.60 ^a^	2.33 ± 0.53 ^a^	2.40 ± 0.58 ^a^	0.457

BMD: Bone mineral density; BMC: bone mineral content. Data are expressed as mean ± standard deviation; T0 to T1: 4-month period without intervention; T1 to T2: 4-month period of zinc supplementation. Different superscript letters indicate significant differences between time points (intragroup analysis) (*p* < 0.05). Test: one-way ANOVA with Bonferroni post hoc test. Low BMD is defined as a z-score ≤ –2 [14].

## Data Availability

The data that support the findings of this study are available from the corresponding author upon reasonable request.

## Supplementary Materials

The following supporting information can be downloaded at: https://www.mdpi.com/article/10.3390/ijerph23060812/s1, Table S1: TREND Statement Checklist.

## Abbreviations

DMD	Duchenne Muscular Dystrophy
BMD	Bone Mineral Density
BMC	Bone Mineral Content
BMI	Body Mass Index
